# Effects of n-3 Polyunsaturated Fatty Acid Supplementation on Cardiovascular Indices in Type 2 Diabetes: A Meta-analysis of Randomized Controlled Trials

**DOI:** 10.31083/RCM25882

**Published:** 2025-02-18

**Authors:** Ruiyao Li, Yao Wang, Jing Xu, Jiahao Yu, Bin Li

**Affiliations:** ^1^School of Clinical Medicine, Shandong Second Medical University, 261053 Weifang, Shandong, China; ^2^School of Clinical Medicine, Shandong First Medical University, 250117 Jinan, Shandong, China; ^3^Department of Cardiology, Jinan Central Hospital, Shandong First Medical University, 250013 Jinan, Shandong, China

**Keywords:** n-3 polyunsaturated fatty acids, type 2 diabetes, cardiovascular indices, meta-analysis

## Abstract

**Background:**

Individuals with type 2 diabetes (T2DM) face a significantly increased risk of cardiovascular disease. This study aims to explore the impact of omega-3 polyunsaturated fatty acids (n-3 PUFAs) on cardiovascular indices in this population. Although the benefits of n-3 PUFAs on cardiovascular health and glycemic outcomes are highly regarded, previous research reports have shown inconsistent results. Therefore, a comprehensive meta-analysis is needed to gain a deeper understanding of the specific effects of n-3 PUFAs on patients with T2DM. To examine the effect of n-3 PUFAs on cardiovascular indices in T2DM using a meta-analysis of randomized controlled trials (RCTs).

**Methods:**

Online databases including PUBMED, EMBASE and Cochrane libraries were searched up to December 2023. We assessed the overall weighted mean difference in cardiovascular indices between the group supplemented with n-3 PUFAs and the control group. The differences were compared uniformly using pre- and post-treatment differences.

**Results:**

Supplementation with n-3PUFAs in patients diagnosed solely with T2DM significantly reduced low density lipoprotein (LDL) (weighted mean difference (WMD) = –3.92, 95% confidence interval (CI) = –6.52 to –1.32, *p* = 0.003 < 0.05), triglycerides (WMD = –23.94, 95% CI = –34.95 to –12.93, *p* = 0.000 < 0.05), cholesterol (WMD = –8.39, 95% CI = –12.06 to –4.72, *p* = 0.000 < 0.05), glycated hemoglobin (WMD = –0.25, 95% CI = –0.41 to –0.06, *p* = 0.003 < 0.05) and the Homeostatic Model Assessment of Insulin Resistance (HOMA-IR) index (WMD = –0.55, 95% CI = –0.81 to –0.29, *p* = 0.000 < 0.05). All other differences in lipid indices, glycemic indices, inflammatory parameters and blood pressure were not statistically significant (*p* > 0.05). Supplementation with n-3 PUFAs decreased high density lipoprotein (HDL) concentration in patients with T2DM and coronary heart disease (CHD) (WMD = –3.92, 95% CI = –6.36 to –1.48, *p* = 0.002 < 0.05). There were no significant differences in LDL, triglycerides, cholesterol, and C-reactive protein (CRP) in patients with T2DM and CHD (*p* > 0.05).

**Conclusions:**

N-3 PUFAs improved lipid levels and long-term blood glucose levels in patients diagnosed solely with T2DM, but did not significantly improve blood pressure inflammatory markers. N-3 PUFAs showed no significant improvement in blood lipid and inflammatory indexes in patients with T2DM and CHD.

**The PROSPERO registration:**

CRD42024522262, https://www.crd.york.ac.uk/prospero/display_record.php?ID=CRD42024522262.

## 1. Introduction

Coronary heart disease (CHD) is a significant and prevalent comorbidity of type 2 diabetes (T2DM), accounting for the majority of diabetes-related deaths [1]. The management of cardiometabolic risk factors—including lipid parameters, inflammatory markers, and blood pressure—has been demonstrated to significantly reduce the risk of CHD and mortality in individuals diagnosed with T2DM [2]. Therefore, it is of paramount importance for individuals with diabetes to implement measures to prevent and manage these risk factors.

Omega-3 polyunsaturated fatty acids (n-3 PUFAs), critical bioactive compounds in various metabolic processes—including α-linolenic acid (ALA), eicosapentaenoic acid (EPA), and docosahexaenoic acid (DHA), have the potential to improve outcomes in patients with diabetes and CHD [3]. Research indicates that a diet enriched with n-3 PUFAs can markedly reduce the risk for cardiovascular disease (CVD) N-3 PUFAs suppress the expression of adhesion molecules in endothelial cells, leading to a decrease in the attachment of leukocytes to endothelial cells as demonstrated by Baker *et al*. [4]. These findings indicate that n-3 PUFAs may reduce leukocyte infiltration into the vessel wall, which could contribute to a reduction in atherosclerosis and a lower risk of cardiovascular disease. N-3 PUFAs also play a role in the improvement of cardiac function. In a rat model of cardiac arrest, Cheng *et al*. [5] found that administering n-3 PUFAs or ascorbic acid at the onset of cardiopulmonary resuscitation (CPR) decreased lipid peroxidation and systemic inflammation, and improved myocardial function and sublingual microcirculation during cardiac arrest and in the initial recovery phase following resuscitation. In addition, a high intake of n-3 PUFAs has a multifaceted impact on the regulation of hyperglycemia and insulin secretion in patients [6]. Wang *et al*. [7] demonstrated that a dose-dependent reduction in plasma levels of n-3 PUFAs, was correlated with decreased glycosylated hemoglobin, type A1c (HbA1c). A daily intake of 0.4 g of n-3 PUFAs was sufficient to lower HbA1c levels to 7% or below in over 95% of patients. However, it has also been reported that n-3 PUFA does not confer any positive impact on glycemia and inflammatory markers in patients [8]. The potential cardiovascular benefits of n-3 PUFAs have been the subject of considerable debate. The diversity of n-3 PUFAs, the wide variety of n-3 PUFAs supplements applied in the trials, the different interventions in the studies, and the differences in efficacy and safety between dietary supplements and prescription drugs may be the main reasons for these conflicting results [9].

In light of the conflicting results of the current clinical studies, we conducted a meta-analysis of the existing randomized controlled trials (RCTs) to assess the impact of n-3 PUFAs on cardiometabolic biomarkers. Our aim was to provide high-quality evidence-based medical evidence of the role of n-3 PUFAs in CVD.

## 2. Methods

### 2.1 Information Retrieval Methodology

This systematic review has been recorded in the PROSPERO database and involved a thorough search of PUBMED, EMBASE, and Cochrane libraries until December 12 2023, using the search strategy detailed in **Supplementary Table 1**. In the case of PubMed, the search formula was as follows: ““Fatty Acids, Omega-3”[Mesh] OR “Eicosapentaenoic Acid”[Mesh] OR “Docosahexaenoic Acids”[Mesh] OR “alpha-Linolenic Acid”[Mesh]” OR “Omega-3” OR “Eicosapentaenoic Acid” OR “EPA” OR “Docosahexaenoic acid” OR “DHA” OR “ω-3” OR “linolenic acid” OR “timnodonic acid” OR “alpha-Linolenic Acid” OR “ALA” OR “omega-3 fatty acid” OR “α-linolenic acid”” AND ““Diabetes Mellitus, Type 2”[Mesh] OR “Diabetes Mellitus, Type 2” OR “Diabetes Mellitus, Noninsulin-Dependent” OR “Diabetes Mellitus, Ketosis-Resistant” OR “Diabetes Mellitus, Ketosis Resistant” OR “Ketosis-Resistant Diabetes Mellitus” OR “Diabetes Mellitus, Non-Insulin Dependent” OR “Diabetes Mellitus, Non-Insulin-Dependent” OR “Non-Insulin-Dependent Diabetes Mellitus”” AND ““Randomized Controlled Trial”[Publication Type] OR “Randomized Controlled Trials as Topic”[Mesh] OR “Controlled Clinical Trial”[Publication Type] OR “randomized controlled trial” OR “RCT” OR “controlled clinical trial””.

### 2.2 Criteria for Eligibility

The following criteria were applied to the studies included in the meta-analysis: (1) The inclusion language was English. (2) The origin of the n-3 PUFA in the trial was limited to fish oil dietary supplements or prescription medications. (3) The study population comprised adult patients diagnosed solely with T2DM or both T2DM and CHD. (4) The outcomes included plasma concentrations of lipoproteins, blood pressure, inflammatory markers, and blood glucose. (5) The trial was limited to double-blind or triple-blind RCTs. (6) There were no date restrictions in this meta-analysis. The following studies were excluded from the analysis: (1) Non-double-blind randomized controlled trials, epidemiological studies, cross-sectional studies, case-control studies animal model studies reviews, abstracts, editorials, and letters. (2) Studies that failed to report on pertinent outcome measures were excluded. (3) Studies lacking access to the full-text or appendix data for assessing methodological quality and data collection were also excluded. (4) Duplicate publications of the same study were discarded.

### 2.3 Data Extraction and Data Evaluation

**Fig. 1.**
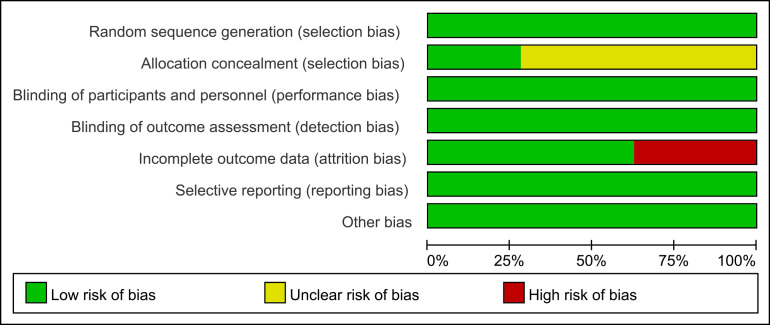
**Risk of bias**.

Two authors conducted individual screening of the titles and abstracts of the studies retrieved through the search strategy to pinpoint studies that aligned with the aforementioned inclusion criteria. Subsequently, the authors conducted a joint evaluation of the full texts of the selected studies to determine their eligibility. All differences were addressed through agreement, and an external party was involved if needed. The data were extracted into standardized spreadsheets and the assessment of the literature’s quality was conducted using a revised Jadad scale. The majority of the studies were of high quality, with 25 articles scoring ≥4 and 7 articles scoring 3. The specific scores are provided in **Supplementary Table 2** below. The quality of the 32 papers included in this study was assessed using the Cochrane Risk of Bias Assessment Scale, as illustrated in Fig. 1 below.

### 2.4 Statistical Analysis

The data was analyzed using STATA 15.0 (StataCorp, College Station, TX, USA) and Review Manager 5.4 (Cochrane Collaboration, Oxford, ENG, UK). The statistical heterogeneity of the included literature was analysed by combining the Q-test and I^2^ test. When *p* > 0.05 and I^2^ < 50%, the heterogeneity was considered to not be significant. Conversely, if *p* ≤ 0.05 and the I^2^ value is greater than 50%, the heterogeneity was considered to be significant. Given the diversity of study designs and populations, random-effects models were used, and sensitivity or subgroup analyses were performed to find sources of heterogeneity. Once the source of the heterogeneity had been identified, subgroup analyses were conducted according to the source. In the event that methodological heterogeneity could not be eliminated, the findings of the combined analyses should be carefully interpreted. For continuous variables, the effect size was determined using the weighted mean difference (WMD). The difference between pre- and post-treatment was used uniformly for comparison. The 95% confidence interval (CI) was used for estimating the interval, and the difference was found to be statistically significant when *p* < 0.05.

## 3. Results

### 3.1 Study Selection

**Fig. 2.**
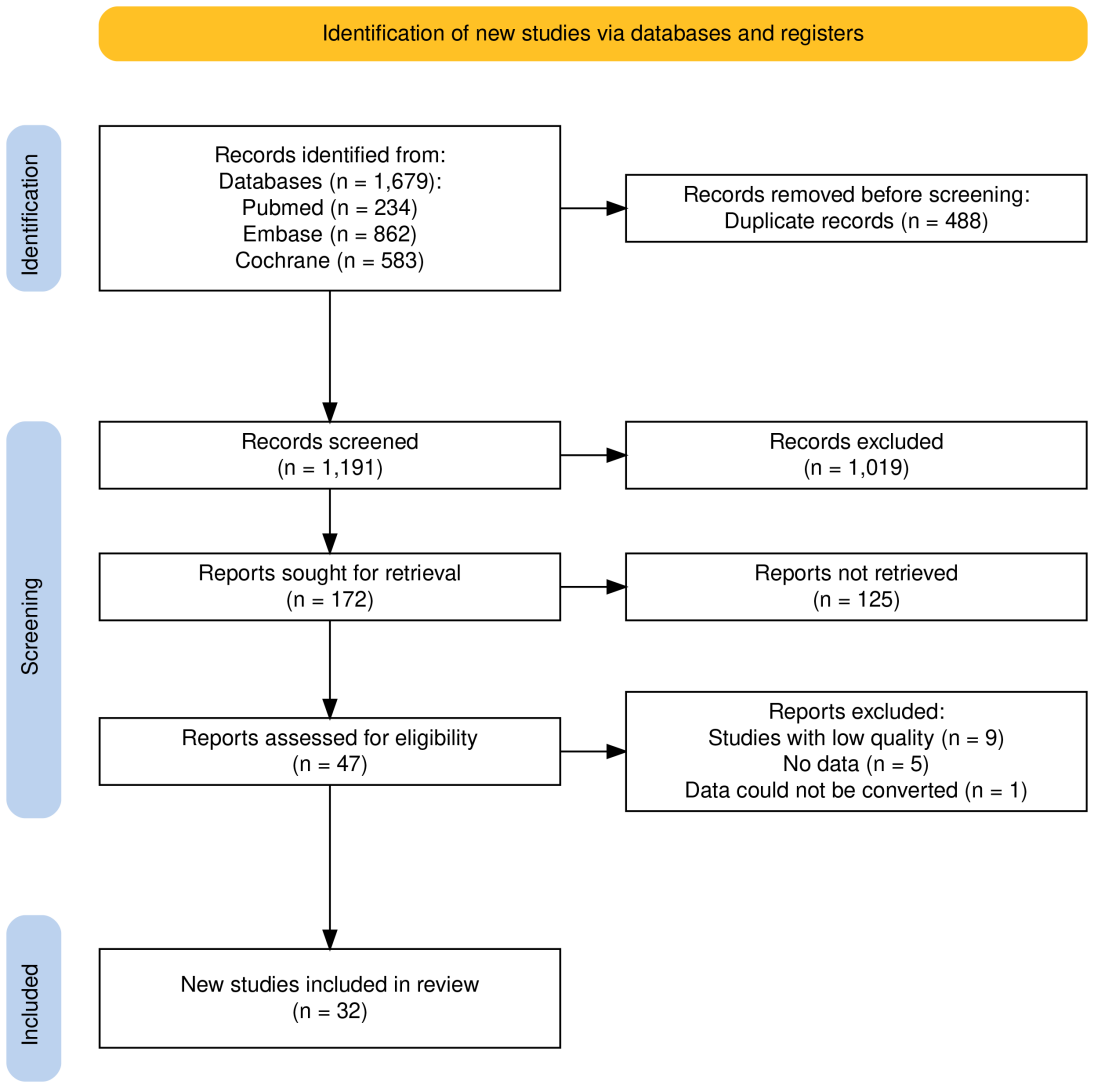
**Flow diagram of study screening**.

The literature search and screening process is illustrated in Fig. 2. 1679 articles were found by searching MEDLINE, EMBASE and the Cochrane Library (PubMed 234, EMBASE 862, Cochrane 583). After removing duplicate literature (488 articles), 1191 articles went through initial screening. After reviewing the titles and abstracts, 1019 articles were excluded, and the remaining ones underwent screening based on the predefined inclusion and exclusion criteria. Ultimately, 32 articles met the eligibility criteria and were included in the analysis.

### 3.2 Study and Participants’ Characteristics

A total of 32 articles satisfied the inclusion criteria since they were published between 1990 and 2023. All studies were double-blind or triple-blind randomised controlled trials. **Supplementary Table 3** provides an overview of the essential characteristics of the literature included and the subgroups of the study population, including age, interventions, duration of follow-up and outcome metrics. The research involved 2046 individuals, with 1128 being placed in the n-3 PUFAs group and 918 in the control group (placebo). Participants were categorized into two groups: 1831 individuals diagnosed solely with T2DM and 215 individuals with confirmed T2DM combined with confirmed CHD. The n-3 PUFA dosage forms employed in the trial group may be broadly categorized as fish oil dietary supplements and prescription n-3 PUFA medications (EPA, DHA, ALA). Of the 32 publications, 62.5% (n = 20) examined the impact of the n-3 PUFA dosage on lipid and lipoprotein profiles, 53.1% (n = 17) on inflammatory parameters, blood pressure, and 84.3% (n = 27) on glycemic control index.

### 3.3 Meta-analysis

#### 3.3.1 Diagnosed Solely with T2DM 

A total of 29 RCTs [10, 11, 12, 13, 14, 15, 16, 17, 18, 19, 20, 21, 22, 23, 24, 25, 26, 27, 28, 29, 30, 31, 32, 33, 34, 35, 36, 37, 38] with 1831 individuals diagnosed solely with T2DM were integrated within the meta-analysis. Supplementation with n-3 PUFAs significantly reduced low density lipoprotein (LDL) (WMD = –3.92, 95% CI = –6.52 to –1.32, *p* = 0.003 < 0.05), triglycerides (WMD = –23.94, 95% CI = –34.95 to –12.93, *p* = 0.000 < 0.05), cholesterol (WMD = –8.39, 95% CI = –12.06 to –4.72, *p* = 0.000 < 0.05), glycated hemoglobin (WMD = –0.25, 95% CI = –0.41 to –0.06, *p* = 0.003 < 0.05) and the Homeostatic Model Assessment of Insulin Resistance (HOMA-IR) index (WMD = –0.55, 95% CI = –0.81 to –0.29, *p* = 0.000 < 0.05). The lipid indices, glycemic indices, inflammatory parameters, and blood pressure did not show statistically significant differences (Table 1). Please refer to the **Supplementary Figs. 1,2,3,4 **for a detailed forest plot.

**Table 1.** **Meta-analysis examining the impact of n-3 PUFA supplementation on cardiovascular indices in patients diagnosed solely with T2DM**.

Variables	No. of studies	Meta-analysis		Heterogeneity	
		WMD (95% CI)	*p* effect	I^2^ (%)	*p* within group
LDL	21	–3.92 (–6.52, –1.32)	0.003	25.2%	0.143 > 0.1
HDL	22	0.69 (–0.24, 1.62)	0.144	77.5%	0.000 < 0.1
Triglycerides	23	–23.94 (–34.95, –12.93)	0.000	86.6%	0.000 < 0.1
Cholesterol	25	–8.39 (–12.06, –4.72)	0.000	57.2%	0.000 < 0.1
DBP	7	–0.13 (–2.32, 2.07)	0.911	25.3%	0.235 > 0.1
SBP	7	1.78 (–1.45, 5.01)	0.280	0.0%	0.679 > 0.1
CRP	6	–0.53 (–1.17, 0.11)	0.106	94.0%	0.000 < 0.1
IL6	3	0.14 (–1.07, 1.36)	0.818	85.9%	0.000 < 0.1
TNF-α	3	–2.62 (–8.43, 3.19)	0.377	63.9%	0.063 < 0.1
Blood glucose	34	–2.63 (–5.77, 0.50)	0.100	75.2%	0.000 < 0.1
Glycated hemoglobin	24	–0.25 (–0.41, –0.06)	0.003	86.6%	0.000 < 0.1
Insulin	18	0.04 (–0.50, 0.42)	0.853	54.1%	0.003 < 0.1
HOMA-IR	14	–0.55 (–0.81, –0.29)	0.000	73.6%	0.000 < 0.1

Note: WMD, weighted mean difference; LDL, low density lipoprotein; HDL, high density lipoprotein; DBP, diastolic blood pressure; SBP, systolic blood pressure; CRP, C-reactive protein; IL6, interleukin 6; TNF-α: tumor necrosis factor α; n-3 PUFA, omega-3 polyunsaturated fatty acid; T2DM, type 2 diabetes; HOMA-IR, Homeostatic Model Assessment of Insulin Resistance.

#### 3.3.2 Diagnosed with T2DM and CHD

A total of 3 RCTs [39, 40, 41] with 215 individuals with confirmed T2DM combined with confirmed CHD were incorporated in the meta-analysis. Supplementation with n-3 PUFAs decreased HDL concentration in patients with T2DM and CHD (WMD = –3.92, 95% CI = –6.35 to –1.48, *p* = 0.02 < 0.05), and no significant differences in LDL, triglycerides, cholesterol, and CRP in patients with T2DM and CHD (*p* > 0.05) (Table 2). Please refer to the **Supplementary Figs. 5,6 **for a detailed forest plot.

**Table 2.** **Meta-analysis showing the effects of n-3 PUFAs supplementation on cardiovascular indices in patients with both T2DM and CHD**.

Variables	No. of studies	Meta-analysis		Heterogeneity	
		WMD (95% CI)	*p* effect	I^2^ (%)	*p* within group
LDL	3	–1.16 (–9.03, 6.71)	0.772	0.0%	0.556 > 0.1
HDL	3	–3.92 (–6.35, –1.48)	0.02	0.0%	0.567 > 0.1
Triglycerides	3	–5.18 (–21.20, 10.84)	0.527	0.0%	0.609 > 0.1
Cholesterol	3	–5.21 (–15.09, 4.68)	0.302	0.0%	0.868 > 0.1
CRP	4	–0.36 (–1.12, 0.39)	0.342	23.2%	0.272 > 0.1

Note: CHD, coronary heart disease.

#### 3.3.3 Sensitivity Analysis

We performed a sensitivity analysis of lipid profiles in patients diagnosed solely with T2DM and found that all the literature analyses were within the 95% CI, and the results were very robust, with no articles found to have a large effect on heterogeneity. Please refer to **Supplementary Fig. 7** for details.

#### 3.3.4 Bias in Publication

**Fig. 3.**
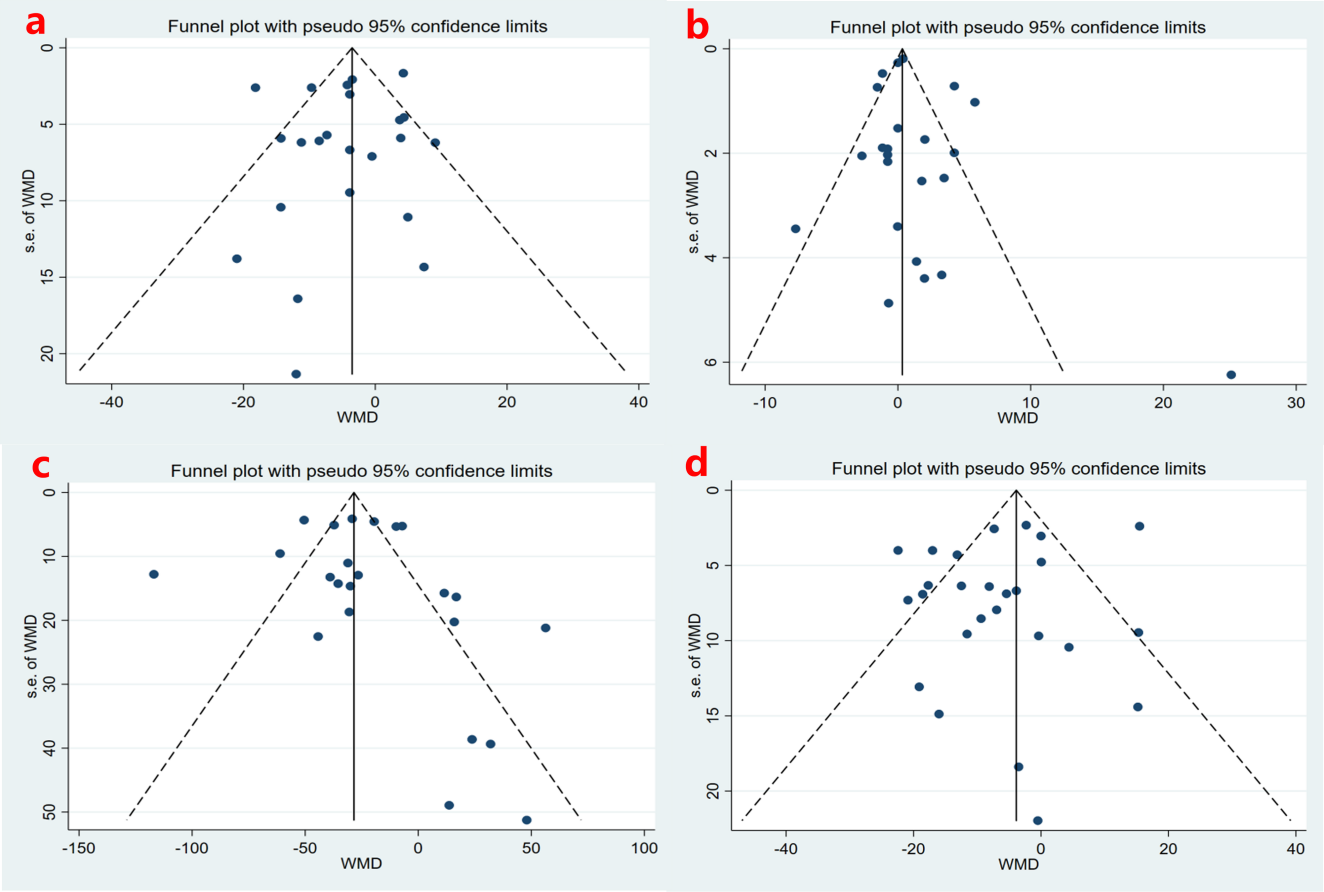
**Funnel plot of the impact of omega-3 polyunsaturated fatty acids (n-3 PUFAs) on lipid indices in individuals diagnosed solely with type 2 diabetes (T2DM)**. (a) LDL; (b) HDL; (c) triglyceride; (d) cholesterol.

The potential for publication bias in patients diagnosed solely with T2DM was evaluated by visually inspecting the funnel plot and conducting the Egger regression test (Fig. 3). All studies fell into the upper part of the funnel diagram and were distributed symmetrically. The selected articles had no obvious selective reporting bias. Egger’s test did not suggest the presence of publication bias (*p* = 0.565, *p* = 0.389, *p* = 0.460, *p* = 0.172). Due to the limited number of studies analyzed and a few studies with small sample sizes, other indicators were not analyzed considering that these plots may not reflect publication bias.

## 4. Discussion

A total of 32 combined RCTs were conducted, involving a total of 2046 adult patients with T2DM. The aim was to evaluate the impact of n-3 PUFAs on cardiometabolic markers in individuals with T2DM and to offer support for the utilization of n-3 PUFA supplementation in averting cardiovascular disease.

In comparison to placebo, n-3 PUFA treatment demonstrated a significant lipid-lowering effect. The mechanism underlying this effect is often attributed to the fact that omega-3 reduces intestinal endogenous cholesterol adsorption and inhibits hepatic cholesterol synthesis. The consumption of n-3 PUFAs led to a decrease in LDL levels, which is consistent with the findings of a previous study [42]. Lowering LDL cholesterol is a key factor in preventing CHD. The primary objective of the American College of Cardiology and American Heart Association is to reduce LDL levels, as it is a major indicator of CHD risk. The administration of LDL-lowering therapies has resulted in a decline in CHD mortality [43]. A reduction in triglycerides was observed in patients with T2DM who consumed n-3 PUFAs, in accordance with previous systematic evaluations [44]. N-3 PUFAs are believed to lower triglyceride levels primarily by decreasing the production of very low-density lipoprotein (VLDL) in the liver. Bornfeldt [45] utilized mouse models and samples of human plasma to uncover an extra mechanism through which these polyunsaturated fatty acids can reduce levels of triglycerides in the bloodstream. The findings indicated that there is a substantial buildup of N-acyl taurine (NAT) in both bile and plasma after the administration of omega-3 supplements. Additionally, the researchers demonstrated that blocking the breakdown of triglycerides in the intestines and the absorption of lipids using NATs (C22:6 NAT) resulted in a reduction in plasma triglyceride levels. Epidemiological research has shown that high levels of triglycerides independently increase the risk of cardiovascular disease. Therefore, the supplementation of n-3 PUFAs may provide a substantial therapeutic advantage in individuals with T2DM by lowering TG levels. The decrease in cholesterol levels and the absence of a substantial rise in HDL following n-3 PUFA consumption does not align with the findings of prior research. Gao *et al*. [46], in their study, discovered that omega-3 cholesterol supplementation did not result in significant overall improvement. However, they noted that HDL levels increased among Asian populations, with the increase seen specifically in the low dose group. The lack of further grouping of the data may be a contributing factor to the inconsistency of the findings.

Previous research has indicated that n-3 PUFAs may elevate HbA1c in patients through mechanisms such as altered hepatic glucose production at rest and reduced insulin release. However, our findings indicated a slight but significant reduction in HbA1c following n-3 PUFA supplementation, this is inconsistent with previous findings Zhang *et al*. [47] who utilized mice with T2DM and treated them by administering Antarctic krill oil orally to demonstrate that omega-3 can decrease inflammation-related factors, particularly tumor necrosis factor α (TNF-α) release. This leads to a reduction in the decrease of insulin signal transduction caused by inflammation-related factors, ultimately alleviating the symptoms of T2DM. In contrast, there was no change in blood glucose, HOMA-IR or insulin levels, which is consistent with the findings of previous study [48]. In a study by Brown *et al*. [48], it was discovered that the increased levels of alpha-linolenic acid, omega-6, and total polyunsaturated fatty acids did not significantly impact glucose metabolism. However, it was noted that there was a potential for an approximate 7% increase in fasting insulin levels with the augmentation of alpha-linolenic acid.

The decrease in plasma concentrations of TNF-α, IL-6, and CRP did not reach statistical significance, aligning with similar outcomes reported in previous research. Inflammation is often responsible for the worst clinical outcomes in patients [49]. Patients who developed T2DM had higher levels of inflammatory markers compared to healthy subjects. Nevertheless, Fayh *et al*. [50] did not observe any significant changes in plasma high-sensitivity C-reactive protein (hs-CRP) levels when they examined post-exercise plasma hs-CRP levels in subjects with T2DM prior to and following the addition of n-3 PUFAs. The primary mechanism by which omega-3 exerts its anti-inflammatory effects is believed to be through the modulation of prostaglandins. The initial hypothesis of a link between omega-3 and cardiovascular protection was based on diets high in marine fats containing omega-3. However, since then the majority of clinical trials have focused on EPA and DHA supplements, and the results of previous studies have not demonstrated conclusive evidence in favor of their beneficial effects. The study findings also revealed no notable alterations in systolic or diastolic blood pressure after the administration of n-3 PUFAs. This discovery aligns with earlier study, indicating that n-3 PUFA supplementation may have had a restricted impact on vascular tone [51].

This meta-analysis represents a relatively comprehensive assessment of 13 cardiometabolic biomarkers. Even though strict inclusion criteria were applied, some limitations must be acknowledged. Another important limitation of the reported trials is the limited number of trials evaluating emerging cardiovascular risk markers as outcomes. It is not possible to combine all identified results, given unstandardized units of measurement and variations in unreported outcomes. As outlined in the PROSPERO protocol, this meta-analysis aimed to evaluate the impact of n-3 PUFAs on those diagnosed solely with T2DM and those diagnosed with T2DM and CHD. Only 3 RCTs studying patients with T2DM combined with CHD met the inclusion criteria. This indicates that a stricter trial design might be necessary, and as a result, the positive outcomes of this analysis could have greater clinical significance.

## 5. Conclusions

In summary, our thorough assessment suggests that n-3 PUFAs enhance lipid profiles in individuals with T2DM. These results are consistent with those of earlier systematic evaluations. However, the improvement in inflammation and glycemia was minimal.

## Availability of Data and Materials

The datasets used and/or analyzed during the current study are available from the corresponding author on reasonable request.

## Supplementary Material

Supplementary material associated with this article can be found, in the online version, at https://doi.org/10.31083/RCM25882.
