# Supplementary material for: Effects of n-3 Polyunsaturated Fatty Acid Supplementation on Cardiovascular Indices in Type 2 Diabetes: A Meta-analysis of Randomized Controlled Trials
Source: Rev Cardiovasc Med. 2025 Feb 18;26(2):25882. doi: 10.31083/RCM25882 (PMC11868883; doi:10.31083/RCM25882)
Supplement: Supplementary file 1 [file 2153-8174-26-2-25882-s1.zip › RCM25882 Supplementary Figures.docx]

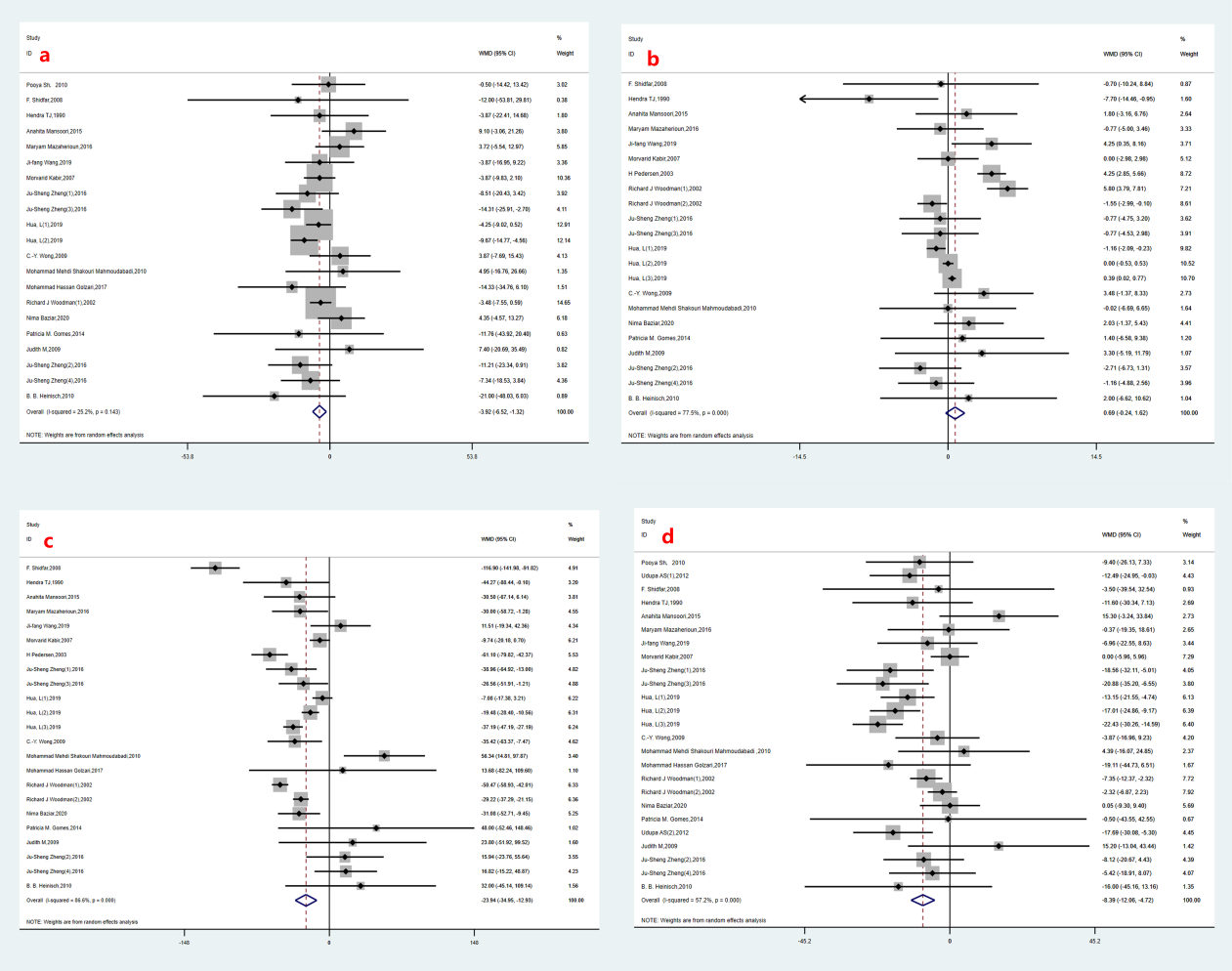


**Supplementary Fig.1** Forest plot of effect sizes for studies assessing the impact of n-3PUFAs on lipid indices amongst patients diagnosed solely with T2DM(a.LDL b.HDL c.triglyceride d.cholesterol)


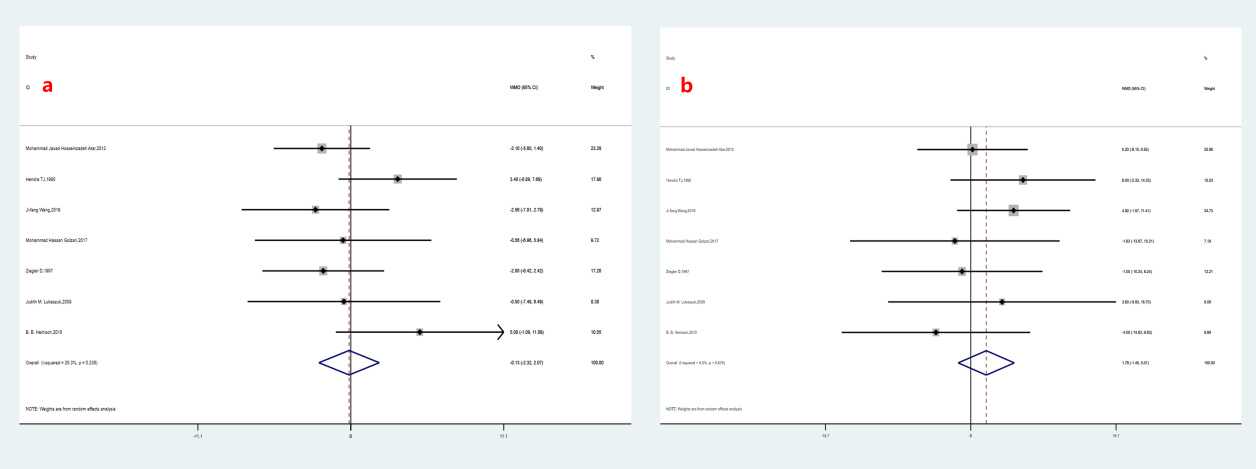


**Supplementary Fig.2** Forest plot of effect sizes displaying the impact of n-3PUFAs on blood pressure amongst adults diagnosed solely with T2DM(a.diastolic blood pressure b.systolic blood pressure)


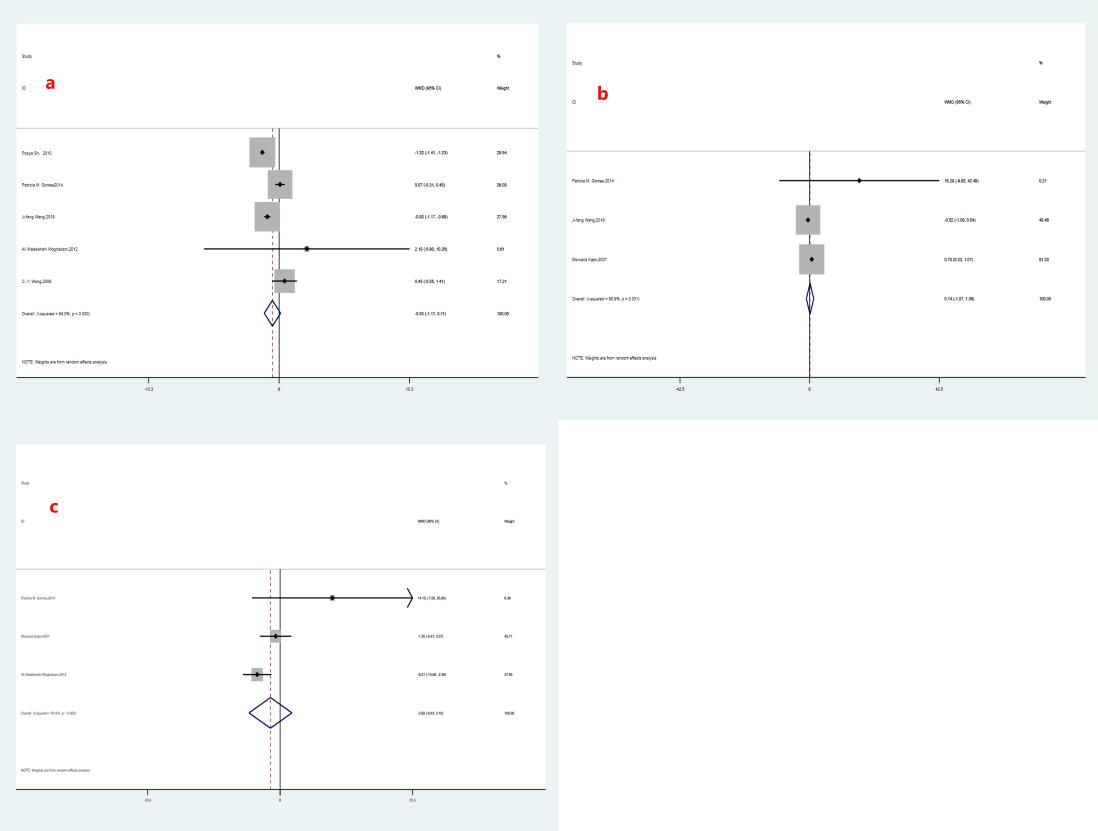


**Supplementary Fig.3** Forest plot of effect sizes displaying the impact of n-3PUFAs on Inflammatory parameters amongst patients diagnosed solely with T2DM(a.CRP b.IL-6 c.TNF-α)


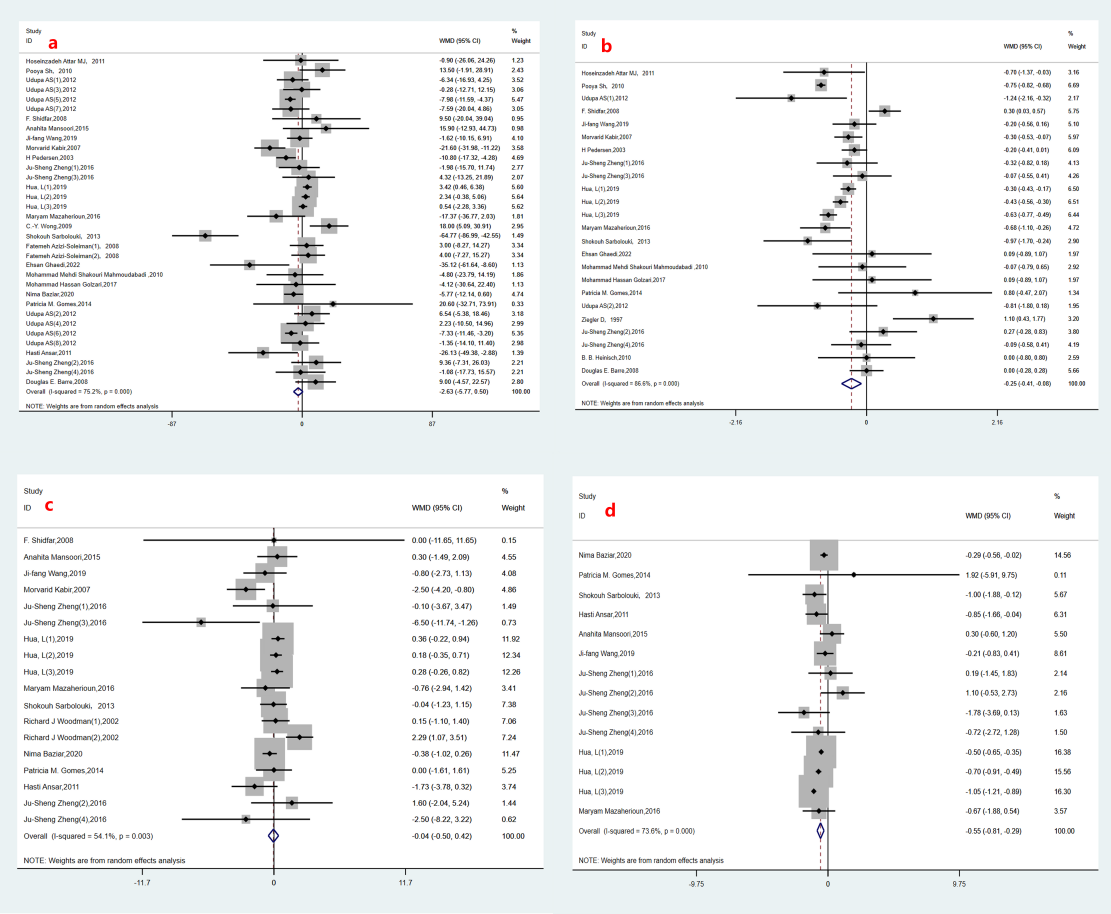


**Supplementary Fig.4** Forest plot of effect sizesfor studies assessing the impact of n-3PUFAs on Indices of glycaemic control levels in individuals diagnosed solely with T2DM(a.blood glucose b.glycated haemoglobin c.insulin d.HOMA-R)


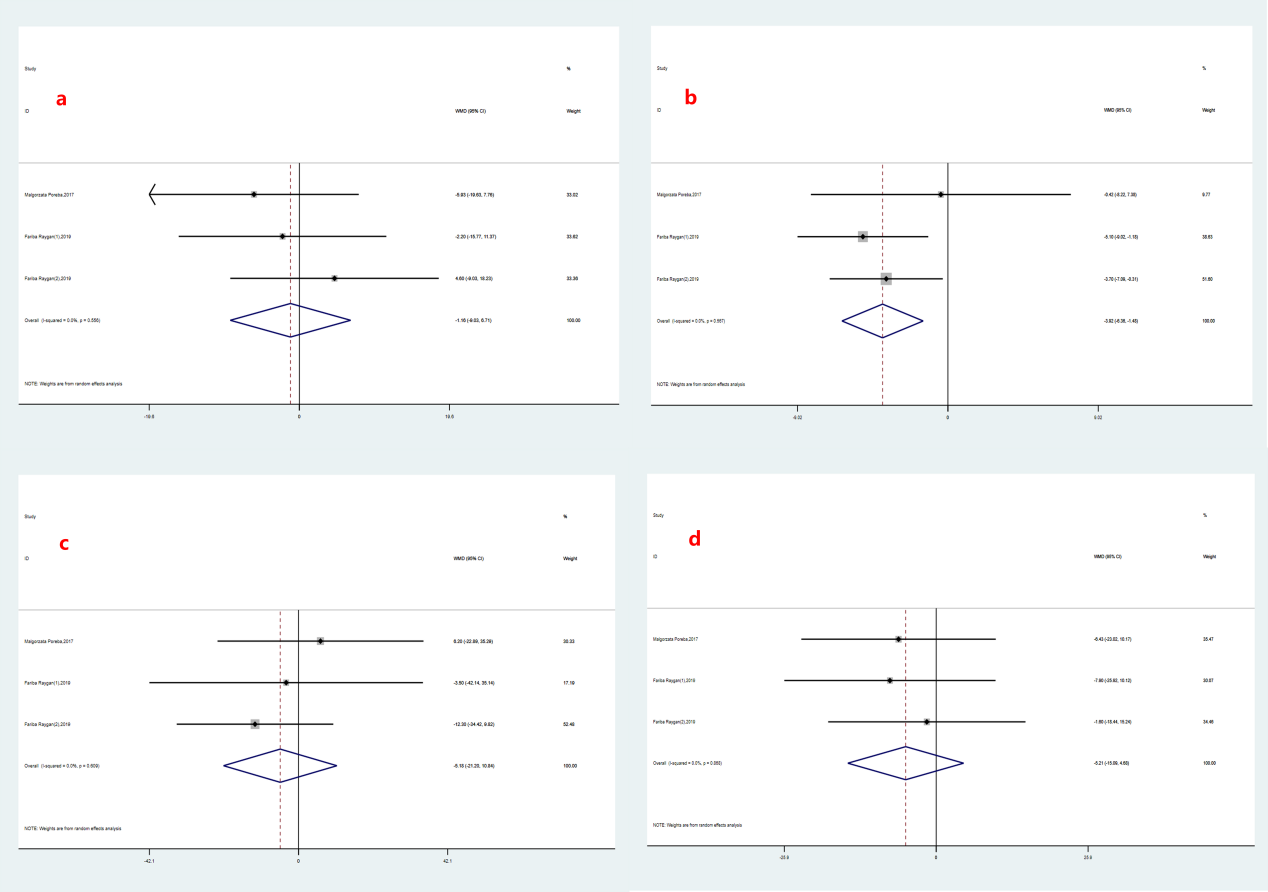


**Supplementary Fig.5** Forest plot of effect sizes for studies assessing the impact of n-3PUFAs on lipid indices amidst adults with T2DM and CHD(a.LDL b.HDL c.triglyceride d.cholesterol)

**
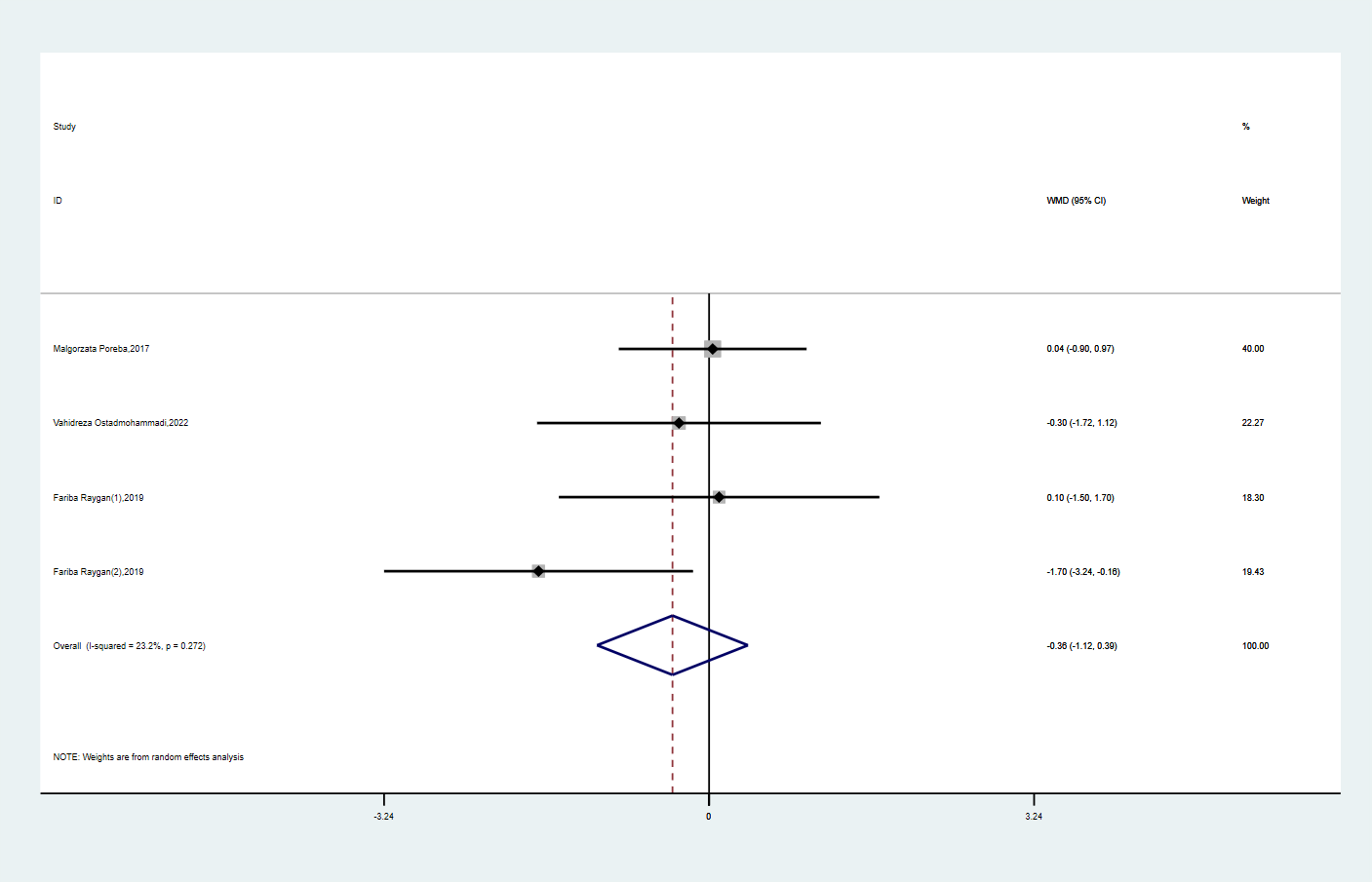
**

**Supplementary Fig.6** Forest plot of effect sizes for studies assessing the impact of n-3PUFAs on CRP amidst adults with T2DM and CHD





**Supplementary Fig.7** Sensitivity analysis used to assess the effect of n-3 polyunsaturated fatty acids on lipid measures in patients diagnosed solely with T2DM(a.LDL b.HDL c.triglyceride d.cholesterol)
