# Supplementary material for: Effects of n-3 Polyunsaturated Fatty Acid Supplementation on Cardiovascular Indices in Type 2 Diabetes: A Meta-analysis of Randomized Controlled Trials
Source: Rev Cardiovasc Med. 2025 Feb 18;26(2):25882. doi: 10.31083/RCM25882 (PMC11868883; doi:10.31083/RCM25882)
Supplement: Supplementary file 1 [file 2153-8174-26-2-25882-s1.zip › RCM25882 Supplementary Tables.docx]

Supplementary Table 1

| **PubMed** | **Search strategy** |
| --- | --- |
| #1 | "Fatty Acids, Omega-3"[Mesh] OR "Eicosapentaenoic Acid"[Mesh] OR "Docosahexaenoic Acids"[Mesh] OR "alpha-Linolenic Acid"[Mesh]" |
| #2 | "Omega-3" OR "Eicosapentaenoic Acid" OR "EPA" OR "Docosahexaenoic acid" OR "DHA" OR "ω-3 " OR "linolenic acid" OR "timnodonic acid" OR "alpha-Linolenic Acid" OR "ALA" OR "omega-3 fatty acid" OR "α-linolenic acid" |
| #3 | #1 OR #2 |
| #4 | "Diabetes Mellitus, Type 2"[Mesh] |
| #5 | "Diabetes Mellitus, Type 2" OR "Diabetes Mellitus, Noninsulin-Dependent" OR "Diabetes Mellitus, Ketosis-Resistant" OR "Diabetes Mellitus, Ketosis Resistant" OR "Ketosis-Resistant Diabetes Mellitus" OR "Diabetes Mellitus, Non-Insulin Dependent" OR "Diabetes Mellitus, Non-Insulin-Dependent" OR "Non-Insulin-Dependent Diabetes Mellitus" |
| #6 | #4 OR #5 |
| #7 | #3 AND #6 |
| #8 | "Randomized Controlled Trial"[Publication Type] OR "Randomized Controlled Trials as Topic"[Mesh] OR "Controlled Clinical Trial"[Publication Type] |
| #9 | "randomized controlled trial" OR "RCT" OR "controlled clinical trial" |
| #10 | #8 AND #9 |
| #11 | #7 AND #10 |

| **Embase** | **Search strategy** |
| --- | --- |
| **#1** | 'omega 3 fatty acid'/exp OR 'bilantin omega' OR 'conchol 36' OR 'eicosa e' OR 'eicosapen' OR 'epaisdin' OR 'epanova' OR 'fatty acids, omega 3' OR 'fatty acids, omega-3' OR 'n 3 fatty acid' OR 'n 3 polyunsaturated fatty acid' OR 'omega 3' OR 'omega 3 carboxylic acid' OR 'omega 3 carboxylic acids' OR 'omega 3 fatty acid' OR 'omega 3 feingold' OR 'omega 3 plus' OR 'omega 3 polyunsaturated fatty acid' OR 'omega forte' OR 'omega-3-carboxylic acids' OR 'omega3 polyunsaturated fatty acid' OR 'sakana' OR 'sanhelios omega 3' OR 'icosapentaenoic acid'/exp OR '5, 8, 11, 14, 17 eicosapentaenoic acid' OR '5, 8, 11, 14, 17 icosapentaenoic acid' OR '5, 8, 11, 14, 17-eicosapentaenoic acid' OR 'eicosa 5, 8, 11, 14, 17 pentaene carboxylic acid' OR 'eicosa 5, 8, 11, 14, 17 pentaenoic acid' OR 'eicosapentaenoate' OR 'eicosapentaenoic acid' OR 'eicosapentenoic acid' OR 'icosa 5, 8, 11, 14, 17 pentaenoic acid' OR 'icosapent' OR 'icosapentaenoate' OR 'icosapentaenoic acid' OR 'omega 3 eicosapentaenoic acid' OR 'unsaturated fatty acid'/exp OR 'ufa' OR 'alkenyl fatty acid' OR 'fats, unsaturated' OR 'fatty acid, unsaturated' OR 'fatty acids, unsaturated' OR 'unsaturated fat' OR 'unsaturated fatty acid' OR 'unsaturated lipid' OR 'cervonic acid'/exp OR '4, 7, 10, 13, 16, 19 docosahexaenoate' OR '4, 7, 10, 13, 16, 19 docosahexaenoic acid' OR 'cervonic acid' OR 'doconexent' OR 'docosa 4, 7, 10, 13, 16, 19 hexaenoic acid' OR 'omega 3 docosahexaenoate' OR 'omega 3 docosahexaenoic acid' |

Continued

| **Embase** | **Search strategy** |
| --- | --- |
| **#2** | 'non insulin dependent diabetes mellitus'/exp OR 'diabetes mellitus, type 2':ab,ti OR 'diabetes mellitus, noninsulin-dependent':ab,ti OR 'diabetes mellitus, ketosis-resistant':ab,ti OR 'diabetes mellitus, ketosis resistant':ab,ti OR 'ketosis-resistant diabetes mellitus':ab,ti OR 'diabetes mellitus, non-insulin dependent':ab,ti OR 'diabetes mellitus, non-insulin-dependent':ab,ti OR 'non-insulin-dependent diabetes mellitus':ab,ti |
| #3 | 'randomized controlled trial'/exp OR 'controlled clinical trial (topic)'/exp OR 'randomized controlled trial (topic)'/exp OR 'controlled clinical trial'/exp |
| #4 | #1 AND #2 AND #3 |

| **Cochrane** | **Search strategy** |
| --- | --- |
| #1 | [Fatty Acids, Omega-3] explode all trees |
| #2 | [Eicosapentaenoic Acid] explode all trees |
| #3 | [alpha-Linolenic Acid] explode all trees |
| #4 | [Docosahexaenoic Acids] explode all trees |
| #5 | ("Omega-3" or "Eicosapentaenoic Acid" or "EPA" or "Docosahexaenoic acid" or "DHA" or "ω-3 " or "linolenic acid" or "timnodonic acid" or "alpha-Linolenic Acid" or "ALA" or "δ-amino linolenic acid" or "omega-3 fatty acid" or "α-linolenic acid"):ti,ab,kw |
| #6 | #1 or #2 or #3 or #4 or #5 |
| #7 | [Diabetes Mellitus, Type 2] explode all trees |
| #8 | (Diabetes Mellitus, Type 2 OR Diabetes Mellitus, Noninsulin-Dependent OR Diabetes Mellitus, Ketosis-Resistant OR Diabetes Mellitus, Ketosis Resistant OR Ketosis-Resistant Diabetes Mellitus OR Diabetes Mellitus, Non-Insulin Dependent OR Diabetes Mellitus, Non-Insulin-Dependent OR Non-Insulin-Dependent Diabetes Mellitus):ti,ab,kw |
| #9 | #7 or #8 |
| #10 | #6 and #9 |

Supplementary Table 2

| Author | Randomization | Allocation concealment | Blinding | Description of withdrawals | total score（max=7） |
| --- | --- | --- | --- | --- | --- |
| Hoseinzadeh Att  ar MJ，2011 | 2 | 2 | 2 | 1 | 7 |
| Pooya Sh，2010 | 1 | 1 | 2 | 1 | 4 |
| Mohammad Javad Hosseinzadeh Atar，2012 | 1 | 1 | 2 | 1 | 5 |
| Nima Baziar,2020 | 1 | 2 | 2 | 1 | 6 |

Continued Supplementary Table 2

| Author | Randomization | Allocation concealment | Blinding | Description of withdrawals | total score（max=7） |
| --- | --- | --- | --- | --- | --- |
| Patricia M. Gomes M.D,2014 | 1 | 1 | 1 | 1 | 4 |
| Udupa AS,2012 | 2 | 2 | 2 | 1 | 7 |
| Shokouh Sarbolouki,2013 | 1 | 2 | 2 | 1 | 6 |
| Ziegler D,1997 | 1 | 1 | 1 | 1 | 4 |
| Fatemeh Azizi-Soleiman,2012 | 1 | 2 | 2 | 1 | 6 |
| F. Shidfar,2008 | 1 | 1 | 1 | 1 | 4 |
| Hendra TJ,1990 | 1 | 1 | 1 | 1 | 4 |
| Ehsan Ghaedi,2022 | 1 | 2 | 2 | 1 | 6 |
| Mohammad Mehdi Shakouri ,2010 | 1 | 1 | 2 | 1 | 5 |
| Judith M. Lukaszuk  ,2009 | 1 | 1 | 1 | 1 | 4 |
| Hasti Ansar,2011 | 1 | 1 | 1 | 0 | 3 |
| Anahita Mansoori,2015 | 1 | 2 | 2 | 1 | 6 |
| Maryam Mazaherioun,2016 | 2 | 2 | 2 | 1 | 7 |
| Ji-fang Wang,2019 | 1 | 1 | 1 | 1 | 4 |
| Morvarid Kabir,2007 | 1 | 1 | 1 | 0 | 3 |
| Mohammad Hassan Golzari,2017 | 1 | 1 | 2 | 1 | 5 |
| H Pedersen,2003 | 1 | 1 | 1 | 0 | 3 |
| Richard J Woodman,2002 | 1 | 1 | 1 | 1 | 4 |
| Ingrid L Mostad,2018 | 1 | 1 | 1 | 0 | 3 |
| Ju-Sheng Zheng,2016 | 2 | 2 | 2 | 1 | 7 |
| Hua,2019 | 1 | 1 | 1 | 1 | 4 |
| B. B. Heinisch,2010 | 1 | 1 | 1 | 0 | 3 |
| Ali Malekshahi Moghadam,2012 | 1 | 1 | 1 | 0 | 3 |
| C.-Y. Wong,2009 | 2 | 2 | 2 | 1 | 7 |
| Douglas E. Barre,2008 | 2 | 2 | 2 | 1 | 7 |
| Malgorzata Poreba,2017 | 1 | 1 | 1 | 0 | 3 |
| Vahidreza Ostadmohammadi,2022 | 2 | 2 | 2 | 1 | 7 |
| Fariba Raygan,2019 | 2 | 2 | 2 | 1 | 7 |

Supplementary Table 3

| Study | No of patients  (Experience/Control) | Age | Treatment vs control | | Follow-up | Endpoint |
| --- | --- | --- | --- | --- | --- | --- |
|  |  | (Experience/Control) |  |  |  |  |
| Hoseinzadeh Att  ar MJ,2011 | 13/34 | 53.6 /53.9 | 720 mg/day EPA+480 mg/day DHA | placebo | 8weeks | 1,2 |
| Pooya Sh,2010 | 40/41 | 56.4/52.7 | 2714 mg/day EPA+DHA | placebo | 8weeks | 1,2,5,8,11 |
| Mohammad Javad Hosseinzadeh Atar,2012 | 39/34 | 53.6/53.9 | 720 mg/day EPA+480 mg/day DHA | placebo | 8weeks | 9 |
| Nima Baziar,2020 | 35/35 | 52.7/52.7 | 1200 mg/day ALA | placebo | 8weeks | 1,3,4,5,6,7,8 |
| Patricia M. Gomes M.D,2014 | 10/10 | 50.1/ 47.0 | 3 g/day ALA | placebo | 8weeks | 1,2,3,4,5,6,7,8,11,12,13 |
| Udupa AS,2012 | 25/25 | 54.9/53.8 | 180 mg/day EPA+120 mg/day DHA | placebo | 4、6、8、12weeks | 1,2,8 |
| Udupa AS,2012 | 25/25 | 53.5/53.8 | 300 mg/day ALA | placebo | 4、6、8、12weeks | 1,2,8 |
| Shokouh Sarbolouki,2013 | 32/35 | 45.0/45.3 | 2 g/day EPA | placebo | 12weeks | 1,2,3,4 |
| Ziegler D,1997 | 39/34 | 57.9/58.6 | 800 mg/day ALA | placebo | 16weeks | 2,9,10 |
| Fatemeh Azizi-Soleiman,2012 | 14/17 | 51.4/56.9 | 1 g/day EPA | placebo | 12weeks | 1 |
| Fatemeh Azizi-Soleiman,2012 | 14/17 | 56.1/56.9 | 1 g/day DHA | placebo | 12weeks | 1 |
| F. Shidfar,2008 | 5/25 | 53.4/54.1 | 2 g/day EPA+DHA | placebo | 10weeks | 1,2,3,5,6,7,8 |
| Hendra TJ ,1990 | 40/40 | 56.0/55.8 | 1 g/day EPA+DHA | placebo | 6weeks | 1,5,6,7,8,9,10 |
| Ehsan Ghaedi,2022 | 18/18 | 44.4/44.7 | 2 g/day EPA | placebo | 8weeks | 1,2 |
| Mohammad Mehdi Shakouri Mahmoudabadi,2010 | 16/17 | 54.2/50.3 | 500 mg/day EPA+200 mg/day DHA | placebo | 8weeks | 1,2,5,6,7,8 |

Continued Supplementary Table 3

| Study | No of patients  (Experience/Control) | Age | Treatment vs control | | Follow-up | Endpoint |
| --- | --- | --- | --- | --- | --- | --- |
|  |  | (Experience/Control) |  |  |  |  |
| Judith M. Lukaszuk  ,2009 | 13/7 | 53.1/56.0 | 600 mg/day ALA | placebo | 12weeks | 2,5,6,7,8,9,10 |
| Hasti Ansar,2011 | 29/28 | 49.0/51.8 | 300 mg/day ALA | placebo | 8weeks | 1,3,4 |
| Anahita Mansoori,2015 | 35/33 | 55.8/ 56.0 | 1450 mg/day EPA+400 mg/day DHA | placebo | 8weeks | 1,3,4,5,6,7,8 |
| Maryam Mazaherioun,2016 | 44/41 | 51.1/50.5 | 600 mg/day EPA+300 mg/day DHA | placebo | 10weeks | 1,2,3,4,5,6,7,8 |
| Ji-fang Wang,2019 | 35/28 | 58.9/56.2 |  | placebo | 12weeks | 1,2,3,4,5,6,7,8,9,10,11,12 |
| Morvarid Kabir,2007 | 12/14 | 55.0/55.0 | 3 g/day EPA+DHA | placebo | 8weeks | 1,2,3,5,6,7,8,12,13 |
| Mohammad Hassan Golzari,2017 | 18/18 | 44.4/44.7 | 2 g/day EPA | placebo | 8weeks | 1,2,5,6,7,8,9,10 |
| H Pedersen,2003 | 23/21 | 63.0/63.0 | 4 g/day EPA+DHA | placebo | 8weeks | 1,2,6,7,8 |
| Richard J Woodman,2002 | 17/16 | 61.2/61.5 | 4 g/day EPA | placebo | 6weeks | 1,2,3,5,6,7,8,9,10 |
| Richard J Woodman,2002 | 17/18 | 60.9/61.5 | 4 g/day DHA | placebo | 6weeks | 1,2,3,5,6,7,8,9,10 |
| Ingrid L Mostad,2018 | 12/14 | 57.0/62.0 | 1.8 g/day EPA+3 g/day DHA | placebo | 8weeks | 1 |
| Ju-Sheng Zheng,2016 | 63/61 | 59.7/59.1 | 2 g/day EPA+DHA | placebo | 12、24weeks | 1,2,3,4,5,6,7,8 |
| Ju-Sheng Zheng,2016 | 63/61 | 59.7/59.1 | 2.5 g/day ALA | placebo | 12、24weeks | 1,2,3,4,5,6,7,8 |
| Hua, L,2019 | 54/59 | 45.6/44.3 | 2 g/day EPA+DHA | placebo | 4、8、12weeks | 1,2,3,4,5,6,7,8 |
| B. B. Heinisch,2010 | 15/15 | 55.0/56.0 | 600 mg/day ALA | placebo | 3weeks | 2,5,6,7,9,10 |
| Ali Malekshahi Moghadam,2012 | 42/42 | 55.4/52.9 | 1548 mg/day EPA+828 mg/day DHA | placebo | 8weeks | 11,13 |

Continued Supplementary Table 3

| Study | No of patients  (Experience/Control) | Age | Treatment vs control | | Follow-up | Endpoint |
| --- | --- | --- | --- | --- | --- | --- |
|  |  | (Experience/Control) |  |  |  |  |
| C.-Y. Wong,2009 | 49/48 | 61.2/59.0 | 4 g/day EPA+DHA | placebo | 12weeks | 1,5,6,7,8,11 |
| Douglas E. Barre,2008 | 18/14 | 59.5/60.7 | 60 mg/kg/day ALA | placebo | 12weeks | 1,2,3 |
| Malgorzata Poreba,2017 | 36/38 | 64.4/66.7 | 2 g/day EPA+DHA | placebo | 12weeks | 5,6,7,8,11 |
| Vahidreza Ostadmohammadi,2022 | 25/26 | 66.1/62.2 | 600 mg/day ALA | placebo | 12weeks | 11 |
| Fariba Raygan,2019 | 30/30 | 64.6/62.0 | 500 mg/day EPA+300 mg/day DHA | placebo | 12weeks | 5,6,7,8,11 |
| Fariba Raygan,2019 | 30/30 | 64.1/62.0 | 400 mg/day ALA | placebo | 12weeks | 5,6,7,8,11 |
